# Sustainable Encapsulation of Cambuci Bioactives via Supercritical-Assisted Atomization

**DOI:** 10.1021/acsomega.6c01444

**Published:** 2026-04-24

**Authors:** Henrique Di Domenico Ziero, Larissa Castro Ampese, Iolanda de Marco, Mariarosa Scognamiglio, Stefania Mottola, Ernesto Reverchon, Tânia Forster-Carneiro

**Affiliations:** † School of Food Engineering, 534751 University of Campinas (UNICAMP), Rua Monteiro Lobato, n.80, Campinas, São Paulo 13083-862, Brazil; ‡ Department of Industrial Engineering, University of Salerno, Via Giovanni Paolo II, 132, Fisciano, Salerno 84084, Italy

## Abstract

The valorization of agro-industrial waste, such as cambuci fruit, is a sustainable strategy to mitigate environmental impacts and add economic value. The goal was to utilize the often-discarded cambuci peel, extract the bioactive compounds, and obtain high-value-added products. The extract was obtained by using a 70% ethanol solution in water. Given the sensitivity to the light and heat of the obtained extract, the supercritical-assisted atomization (SAA) technique was used for encapsulation, varying the extract to PVP ratios (1:2, 1:5, 1:10, and 1:15). The SAA system involved mixing liquid CO_2_ and the extract/PVP, followed by atomization in a precipitation chamber. The best results obtained in this study were achieved with an extract-to-PVP ratio of 1:15, which yielded the highest value (72%) and the highest levels of antioxidant capacity (142.83 μmol of Trolox/g of cambuci) and total phenolic compounds (69.94 mg of GAE/g of cambuci). Furthermore, the particles produced at this ratio had an average size of 1.17 μm with a relatively homogeneous size distribution. Moreover, after 5 months, the encapsulated cambuci exhibited better conservation of phenolic compounds and antioxidant activity compared with the cambuci extract stored under the same conditions. The use of Path2Green showed that this production process more closely adheres to green chemistry principles compared with two similar processes documented in the literature. These findings demonstrate that encapsulation with PVP, achieved through the SAA process, effectively preserves bioactive compounds and produces spherical, stable particles suitable for use in the nutraceutical and pharmaceutical industries, aligning with sustainability goals.

## Graphical abstract

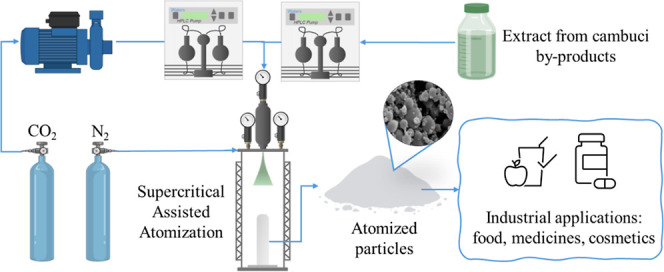

## 1 Introduction

Food and agricultural waste, mainly biomass, also represents a problem and an opportunity. On the one hand, it poses an environmental risk with potential pollution, greenhouse gas (GHG) emissions, and financial losses for companies. On the other hand, it could also represent a business opportunity, a new energy source, or a source of different bioactive compounds. That is the difference between a linear economy and a circular economy perspective. Vegetal waste is one of the most significant wastes on a worldwide scale, consisting of approximately one-third of the material that enters the industry,^1^ and the agricultural and food sectors contribute to approximately 25% of the total GHG emissions.^2^

*Campomanesia phaea* O. Berg is a native Brazilian plant of the Myrtaceae family that produces a small fruit called cambuci, rich in polyphenols such as ellagitannins and proanthocyanidins^3^ and with a good antioxidant activity.^4^ Local producers use the fruit to make juices, ice creams, and alcoholic drinks. However, the cambuci peel, which represents around 18% of the fruit’s mass and is a source of phenolic compounds,^5^ does not have a definitive destination due to the scale of production, and producers usually take the peel for composting. Jimenez Moreno et al. (2024) studied the extraction and hydrolysis of cambuci peel with subcritical water, managing to extract polyphenols and sugars from the peel with real-time detection of the compounds.^6^ Nevertheless, a key challenge with this extract is its sensitivity to environmental factors and quick breakdown under sunlight. To preserve their biological properties, it is necessary to encapsulate them with a protective agent.

Encapsulation technologies have been widely used to protect compounds from environmental degradation, facilitate storage, and increase the bioavailability of compounds.^6–8^ Due to the urgent need to adopt more ecofriendly methods consistent with green chemistry principles, supercritical-assisted atomization (SAA) can be used to microencapsulate an extract within microparticles employing a polymer as an encapsulation component. Despite its efficiency, SAA has some limitations, for example, the solubility of solvents and polymers in CO_2_.

Polyvinylpyrrolidone (PVP) was primarily chosen as the polymer to encapsulate the extract because of its FDA approval, extensive pharmaceutical applications, and biocompatibility. It serves as an effective carrier for coprecipitation, maintaining the biological activity of delicate compounds.^9^

In some previous work, Capua et al.[Bibr ref10] (2019) performed an SAA of β-carotene to produce PVP-β-carotene microparticles to increase the solubility and bioavailability of β-carotene with two different solvents: ethanol and acetone/ethanol 70/30 (v/v). It was reported that microparticles had a spherical shape, with the particle size varying for each solvent used, and were able to preserve the antioxidant capacity of β-carotene.^10^

SAA is an advanced encapsulation method that utilizes carbon dioxide under supercritical conditions as an atomizing agent. This technique allows for the production of dry particles within a single phase, operating at moderate temperatures. It also markedly decreases the use of organic solvents, thereby enhancing the environmental profile and overall quality of the final product.^11^

Implementing sustainable strategies in chemical process design and development is becoming more crucial in the field of contemporary chemistry. The 12 principles of Green Chemistry serve as a theoretical and practical guide for minimizing environmental harm, improving process efficiency, and promoting renewable raw materials and clean technologies.

The Path2Green approach is presented as a tool to assess a process’s conformity to green chemistry principles, providing a quantitative and comparative view of its sustainability in comparison with other processes.^12^

Encapsulating natural extracts, particularly from residual plant biomass, such as fruit leaves, offers a way to valorize renewable resources within a circular economy. Nevertheless, conventional methods for stabilizing and delivering bioactive compounds frequently rely on organic solvents and energy-heavy processes, potentially compromising overall sustainability. Considering this, the present study aimed to develop microparticles containing the ethanolic extract of cambuci bagasse (peel) encapsulated with PVP as a polymer. Different extract-to-PVP ratios were tested to optimize the encapsulation process and to protect bioactive compounds from environmental factors, such as oxidation, light, and temperature. This approach aims to enhance the stability and longevity of phenolic compounds and their antioxidant activity, thereby ensuring their preservation over time for potential applications in the nutraceutical, pharmaceutical, and cosmetic industries. Moreover, particular attention was paid to the evaluation of the sustainability of the process in light of the criteria defined by Path2Green.

## 2 Materials and Methods

### 2.1 Materials

Cambuci bagasse was donated by Asmussen Company (Natividade da Serra, SP, Brazil). The bagasse was then dried at 30 °C in an oven overnight, milled in a blender, and sieved through a 3 mm sieve to obtain a uniform-sized bagasse. Folin–Ciocalteu reagent (code: TC54995PP) was supplied by TITOLCHIMICA S.p.A. DPPH reagent (code: D913-2) was purchased from Sigma-Aldrich (Merck KGaA, Darmstadt, DE). Tetrahydrofuran, trifluoroacetic acid, acetonitrile, and absolute ethanol were purchased from Carlo Erba (Milan, Italy). The distilled water was produced by using a lab-scale plant in our laboratories.

### 2.2 Vegetal Compound Extraction and Main Composition

Cambuci bagasse is mainly composed of 9.41% moisture, 3.18% ashes, 7.31% fats, 4.93% protein, and 75.17% sugars and fibers.^13^

Different extraction methods were tested based on the literature: 70/30 ethanol/water, 80/20 ethanol/water, and 70/30 methanol/water solutions (v/v). Cambuci bagasse powder was mixed with the extraction solution in a proportion of 1 g/10 mL and left in the dark for 24 h under agitation at ambient temperature (∼25 °C). It was then centrifuged for 30 min at 5300*g* and 4 °C to remove excess powder. The solution was sequentially filtered through a vacuum pump with a 0.1 μm paper filter to remove all of the impurities. The phenolic-rich extract was stored in the dark at 4 °C in a freezer for subsequent utilization.

### 2.3 Supercritical-Assisted Atomization

The ethanolic cambuci extract was used in the SAA process, with varying ratios of cambuci extract and PVP: 1:2, 1:5, 1:10, and 1:15 (extract mass/PVP mass). Table 1 presents the experimental setup.

**Table 1:** Experimental Parameters Used in the System^a^

	mass ext (g)	mass PVP (g)	T1 (C°)	T2 (C°)	T3 (C°)	TA (C°)	PM (bar)	QLC (liq/CO_2_)	Q N_2_ (mL/min)
SAA 1:2	0.69	1.38	90	100	100	130	120	1.54	1200
SAA 1:5	0.789	3.94	90	100	100	130	120	1.52	1200
SAA 1:10	0.46	4.6	90	100	100	130	120	1.61	1200
SAA 1:15	0.23	3.45	90	100	100	130	120	1.66	1200

a MASS EXT: mass of cambuci extract in the solution pumped to the system; T1: temperature in the mixer; T2: temperature in the middle chamber; T3: temperature in the filter region; TA: temperature of the gaseous nitrogen (N_2_); PM: average pressure measured in the mixer during the procedure; QLC: ratio between the liquid flux and the CO_2_ flux pumped in the system; Q N_2_: flux of gaseous nitrogen pumped to the system.

The apparatus used for SAA consists of two high pressure pumps (model 305 RECON, Gilson, USA), one for pumping the liquid CO_2_ and the other for the liquid substrate, and a cooler (model ecoline RE106, Lauda, USA) to secure the CO_2_ temperature of −10 °C and the liquid state. This saturator consists of a high-pressure vessel of 25 cm^3^ filled with stainless steel perforated saddles to create a high contact surface chamber for properly mixing the CO_2_ and the substrate. The resultant solution is sprayed through a thin-wall injector nozzle with an internal diameter of 80 μm to a precipitation vessel of 3 dm^3^. Additionally, N_2_ was heated by using electrical resistance (model CBEN 24G6, Watlow, USA) before being introduced into the precipitation vessel to facilitate droplet evaporation. The saturator and precipitation vessel are constantly heated with electrical resistances (model STB3EA10, Watlow, USA). A stainless-steel filter is located on the bottom of the precipitation vessel to separate the formatted powder from the gas streamflow (CO_2_, solvent, and N_2_). Figure 1 illustrates the SAA system. The process yield was calculated considering the mass of PVP and cambuci extract applied in the SAA process and the mass recovered in the filter.

**Figure 1:**
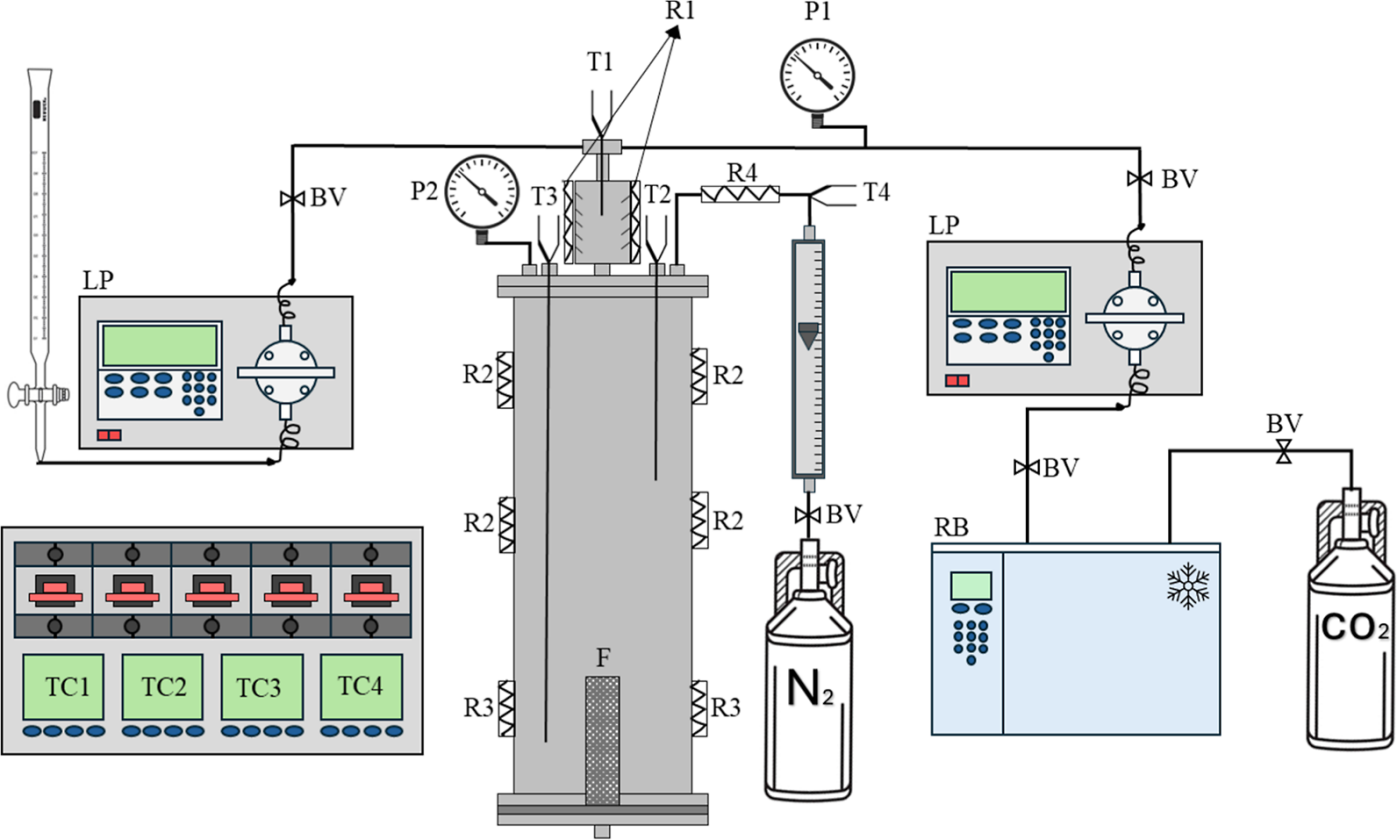
Schematic illustration of the SAA system, where BV is block valve; P1 is the pressure in the mixer; P2 is the pressure in the chamber; T1, T2, T3, and T4 are the thermocouples to measure the temperature in the mixer, middle chamber, near the filter, and of the azote line, respectively; TC1, TC2, TC3, and TC4, are the temperature controllers of the resistances R1, R2, R3, and R4, based on the temperature signal of T1, T2, T3, and T4, correlated by the indicative number. F is the filter; LP(s) are the liquid pump(s); RB is the refrigerated bath.

### 2.4 Morphology and Particle Size

Sample images and structures were obtained through analysis using a field emission scanning electron microscope (model Supra 35, Carl Zeiss SMT AG, Oberkochen, Germany). To perform this, the powder sample was fixed on a carbon tab (Agar Scientific, Stansted, UK) and coated with a thin layer of gold (250 Å) using a sputter coater (mod. B7341, Agar Scientific). Several SEM photomicrographs were taken from different parts of the sample, for each experimental run, to verify the conformity of the powder. The photomicrographs were used to measure particles’ size using the Sigma Scan Pro Software (release 5.0, Aspire Software International, Ashburn, VA), and around 600 microparticles of each treatment were counted. After that, the data obtained from the particle size were analyzed in the Origin software (release 8.5.1, OriginLab, Massachusetts, USA) to produce histograms of particles’ size.

### 2.5 Antioxidant Capacity

The antioxidant capacity was measured by DPPH radical scavenging activity, adapted from Brand-Williams et al.^14^ A Trolox standard curve was prepared to quantify the antioxidant activity in terms of μM Trolox equivalent (eq). The DPPH solution was prepared with the DPPH reagent and ethanol. The cambuci extract was freeze-dried and resuspended in ethanol, and the encapsulated extract was dissolved in ethanol. 2.925 mL of the DPPH solution was pipetted into cuvettes with 75 μL of the samples dissolved in an ethanolic solution, stored in the dark for 30 min, and read in a UV/vis spectrometer (model Cary60, Agilent Technologies, USA) at 515 nm. The Origin software (release 8.5.1, OriginLab, Boston, MA, USA) was used to plot the results. Results are expressed in μM Trolox eq/g dry cambuci in Table 2.

**Table 2:** Characterization of the Particles Formed in the SAA Process, Antioxidant Activity, and Phenolic Compounds^b^

	yield (%)	antioxidant capacity	total phenolic compounds	ave. size	*D*_10_	*D*_50_	*D*_90_	PSD
cambuci extract	-	198.78 ± 12.63^a^	77.83 ± 1.39^a^	-	-	-	-	-
SAA 1:5	43	85.20 ± 3.11^a^	57.90 ± 2^a^	0.51	0.35	0.68	1.52	1.72
SAA 1:10	54	107.55 ± 0.39^a^	60.75 ± 1.82^a^	0.91	0.54	0.91	1.68	1.25
SAA 1:15	72	142.83 ± 7.38^a^	69.94 ± 3.52^a^	1.17	0.54	1.19	2.75	1.85

a Results differ from each other at the 0.05 significance level.

b PSD means particle size distribution; *D*_10_, *D*_50_, and *D*_90_: particle size percentiles (10%, 50%, and 90% of particles below the specified diameter).

### 2.6 Total Phenolic Compounds

The total phenolic compounds were measured by the adapted Folin–Ciocalteu spectrophotometric method.^15^ The Folin–Ciocalteu reagent was used to prepare a 3% reagent solution. In 3 mL glass cuvettes, 0.25 mL of extract and 2.75 mL of the 3% Folin–Ciocalteu solution were added, and the mixture was placed in the dark for 5 min. After that, 0.25 mL of the 1 M Na_2_CO_3_ solution was added to the mix. The final mixture was placed in the dark for 1 h, and sequentially, the absorbance was read in a UV/vis spectrometer at 765 nm. A gallic acid standard curve was made to calculate the concentration of total phenolic compounds in gallic acid equivalent (GAE). The Origin software (release 8.5.1, OriginLab, Massachusetts, USA) was used to plot the results. Results are expressed in mgGAE g^–1^ of the dry matrix, as shown in Table 2.

### 2.7 High-Performance Liquid Chromatography Analysis

To identify compounds in the samples produced by extraction and after the SAA process, an HPLC (Agilent, USA) coupled with a DAD detector was used. To perform the analysis, the samples were resuspended in methanol. The HPLC was equipped with a Prodigy ODS3 C18 column (250 × 4.60 mm, 5 μm), and the flow rate was set at 0.8 mL/min to perform the separation process. Two solvents were used: A and B. Solvent A was a mixture of 98:2:0.1 v/v of H_2_O, tetrahydrofuran, and trifluoroacetic acid, respectively, and solvent B was acetonitrile. The gradient of solvents was set initially to 91% A for 5 min, to 84% at 15 min, then to 70% at 35 min, and finally to 91% again at 40 min, based on the literature.^4,16^ The standard curves of ascorbic acid (AA) and gallic acid were prepared. Results were obtained in peaks of compound concentration and converted to milligrams of the compound to g of the extract using the standard curve.

### 2.8 Complex PVP Extract Protection Capability

The extract and the SAA result were stored in a translucent vial and left at room temperature and in light to simulate common storage conditions. The vial was hermetically closed to avoid humidity from entering. After five months, total phenolic compounds were evaluated by the Folin–Ciocalteu spectrophotometric method, and antioxidant capacity was measured by DPPH radical scavenging activity (described in Sections 2.4 and 2.5).

### 2.9 Sustainability Metric Path2green

The sustainability score was obtained using the Path2Green methodology.^12^ Two other studies were selected, and the methodology was applied to compare the score with other studies. Path2Green was developed based on the 12 principles of green chemistry established in 1998.^17^

### 2.10 Statistical Analysis

The data from total phenolic compounds and antioxidant activity were submitted to analysis of variance (ANOVA) with a significance level of 0.05 and the Tukey test.

## 3 Results and Discussion

The results obtained in this study provide an understanding of the encapsulation of the cambuci extract in PVP microparticles using the SAA technique. PVP was effective in encapsulating the cambuci extract at concentrations of 1:5, 1:10, and 1:15. The analysis is structured to address the key findings related to the encapsulation process, particle morphology, antioxidant activity, preservation of phenolic compounds, and stability of bioactive compounds over time. The findings are analyzed, highlighting the potential of SAA as a viable method for preserving and enhancing the bioactive properties of natural extracts for applications in distinct industries. The main results of the experiment are summarized in the table below (Table 2) and discussed in the sequential topics.

The extraction tests resulted in a better performance of 70% methanol solution, resulting in 12.55% extract yield, followed by 70% ethanol (11.74% yield) and 80% ethanol with 9.17%. 70% ethanol was chosen as the extraction solvent due to its similar performance to the methanol solution and environmental and human safety.

### 3.1 SAA Result and Morphology

Figure 2 presents the SAA result (Figure 2A) and the SEM images obtained (Figure 2B,C). It is possible to note in Figure 2B the spherical formation of the PVP-extract complex due to the complete coating of the cambuci extract. The SAA 1:5, 1:10, and 1:15 worked properly, resulting in a reasonable amount of powder, ranging from 43 to 72% of the initial mass used in the procedure (mass of dry extract + mass of PVP), and the amount of powder recovered increased with the fraction of PVP added to the process. Due to the characteristics of the internal surface of the chamber and filter, it is very difficult to recover all of the powder produced. This indicates that a minimum amount of lost mass is expected, and once this amount has been exceeded, the yield of the recovered powder will increase. SEM images showed that almost all of the particles produced presented a spherical shape.

**Figure 2:**
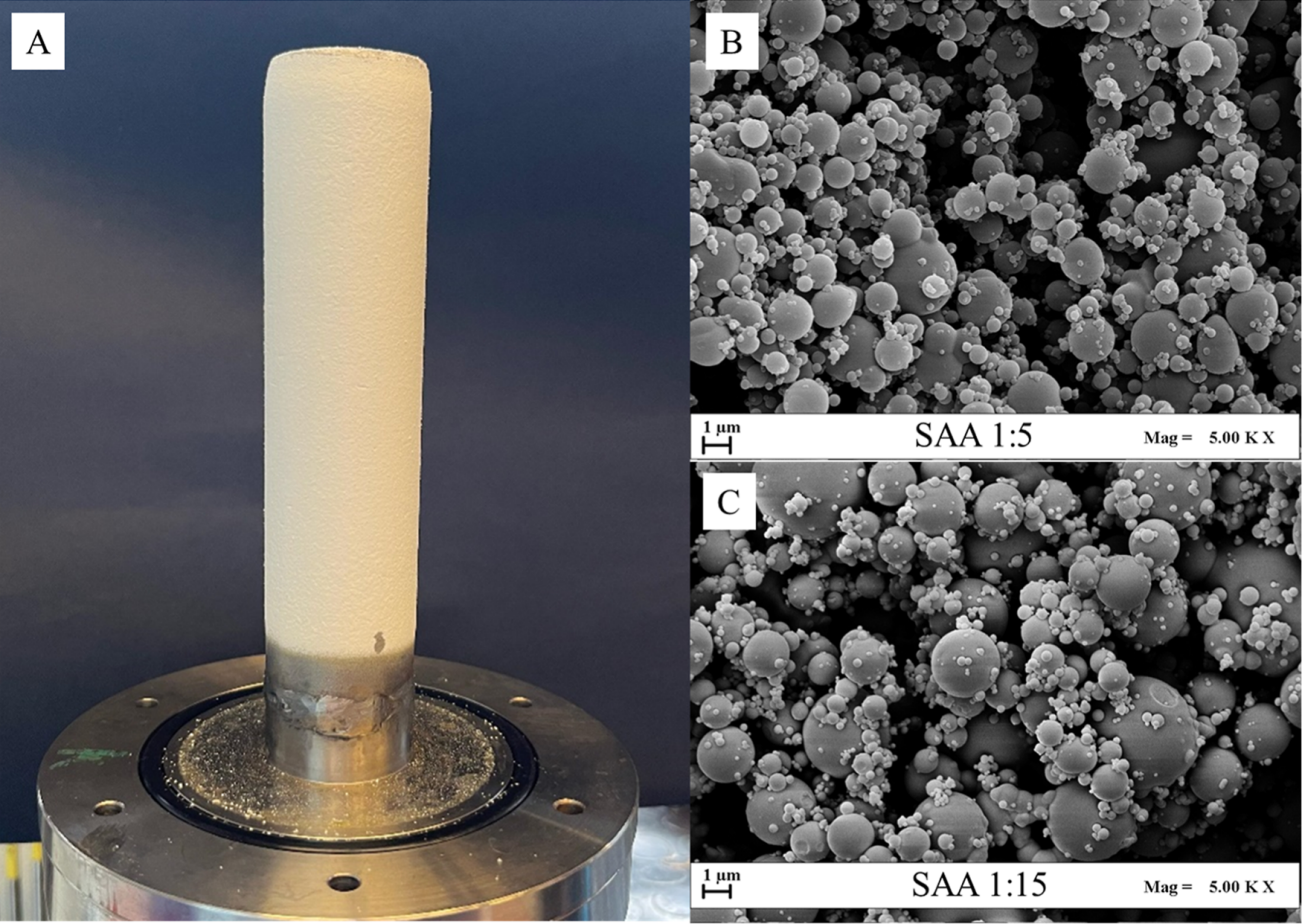
Encapsulated cambuci extract in PVP particles held by the filter (A) after the procedure and SEM of SAA 1:5 (B) and 1:15 (C).

The SAA 1:2 procedure did not yield sufficient powder for posterior analysis, and the SEM images indicate that the process did not occur properly due to the formation of agglomerates with irregular shapes. Probably, there was not enough PVP to coat the extract particles completely and form a spherical structure. The irregular complex also formed links with other structures, resulting in large agglomerations. Similar results with lower PVP ratios have been reported in the literature for different compounds. For instance, Duta Lestari et al.^18^ worked with Curcuma extract and found that a 1:10 extract to PVP ratio resulted in irregular particle formation and agglomeration. Their results were supported by Chhouk et al.^19^ that found the same situation with curcumin in a ratio of 1:10 compound to PVP. This indicates that PVP performs an important role in the formation of microparticles in supercritical conditions and may be a minimum amount of PVP to produce regular particles, which probably vary according to the compound/extract being processed.

Figure 3 presents the distribution curves of particle sizes. Particle size tended to decrease when a higher PVP fraction was added to the process. SAA 1:15 has the biggest particle size with an average diameter of 1.17 μm, SAA 1:10 has 0.91 μm, and SAA 1:5 has the smallest with 0.51 μm. The particle size behavior is associated with the properties of the extract and encapsulation agent, such as concentration and viscosity.^20^ A possible reason for this behavior is that more cambuci-PVP particles were formed instead of increasing the particle size. Another possible reason is that due to the large amount of PVP present in the SAA 1:15 system, more empty particles were formed. The polydispersity index (PSD), which measures the variation in particle sizes after the process and indicates the particle’s homogeneity, also decreased with the increase of PVP in the system (Table 2). Specifically, 1:10 SAA yielded the lowest PSD result of 1.25. Lower PSD results indicate that the particles have a more homogeneous size distribution.^14^ More homogeneous particles are desired for practical applications, such as the nutraceutical or medical industry, because they have the same release ratio and diffusivity behavior.^21^

**Figure 3:**
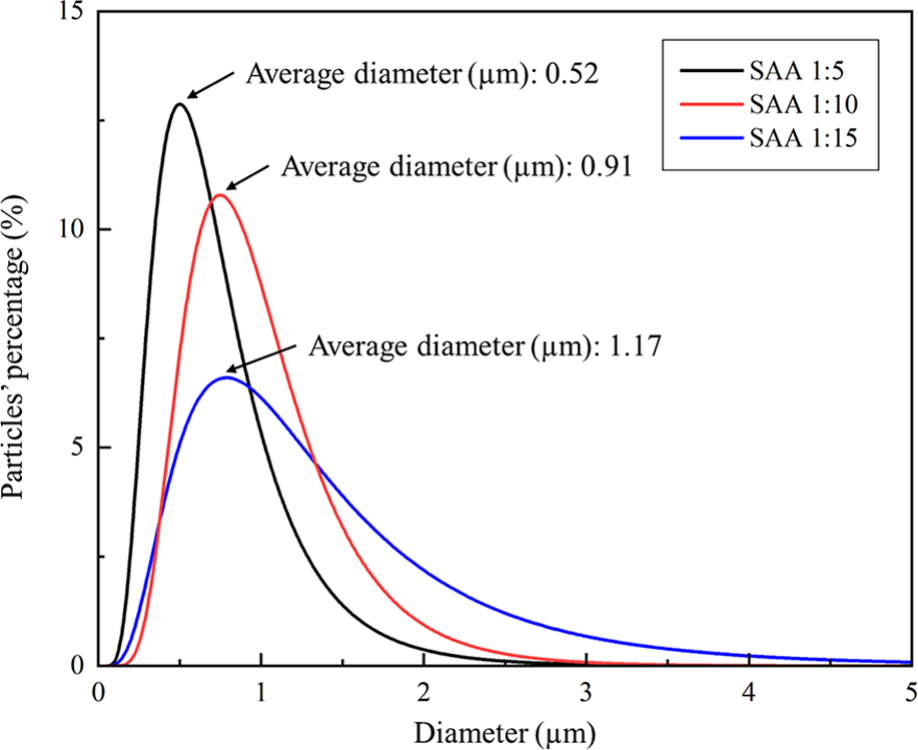
Fitting line of the particles’ size for SAA 1:5 in black, SAA 1:10 in red, and SAA 1:15 in blue.

### 3.2 Antioxidant Activity and Phenolic Compounds

The preservation of the phenolic compounds and the antioxidant capacity of the cambuci bagasse are the main objectives of this study. Both are important components for human health, reducing the risk of diseases and improving the quality of life.^16,22,23^ Additionally, they serve as ingredients used as additives in the nutraceutical, pharmaceutical, and cosmetic industries.^24,25^

Figure 4 presents the phenolic compounds and antioxidant capacity of the cambuci extract and SAA products based on dry cambuci bagasse. ANOVA results indicate that each treatment is significantly different from the others at a significance level of 0.05. Cambuci bagasse presented 77.83 mg GAE/g of dry matter (or 7.783 mg GAE/ml) for total phenolic compounds and 198.78 μm Trolox eq/g of dry matter (both presented in Table 2). The values are higher than those found in the literature for the cambuci fruit, which varied from 2.4 to 4.4 mg GAE/mL^16,26–28^ but no value was found in the literature for cambuci bagasse or cambuci peel. Some fruits have more phenolic compounds in the peel than in the pulp; that is the case of pomegranate,^29^ apple,^30^ and orange,^31^ for example. The values obtained for antioxidant activity were 85.2 μmol trolox/g cambuci, 107.55 μmol trolox/g cambuci, and 142.83 μmol trolox/g cambuci for SAA 1:5, 1:10, and 1:15, respectively. The total phenolic compounds were 57.9, 60.75, and 69.94 mg of GAE/g of cambuci, respectively, for SAA 1:5, 1:10, and 1:15.

**Figure 4:**
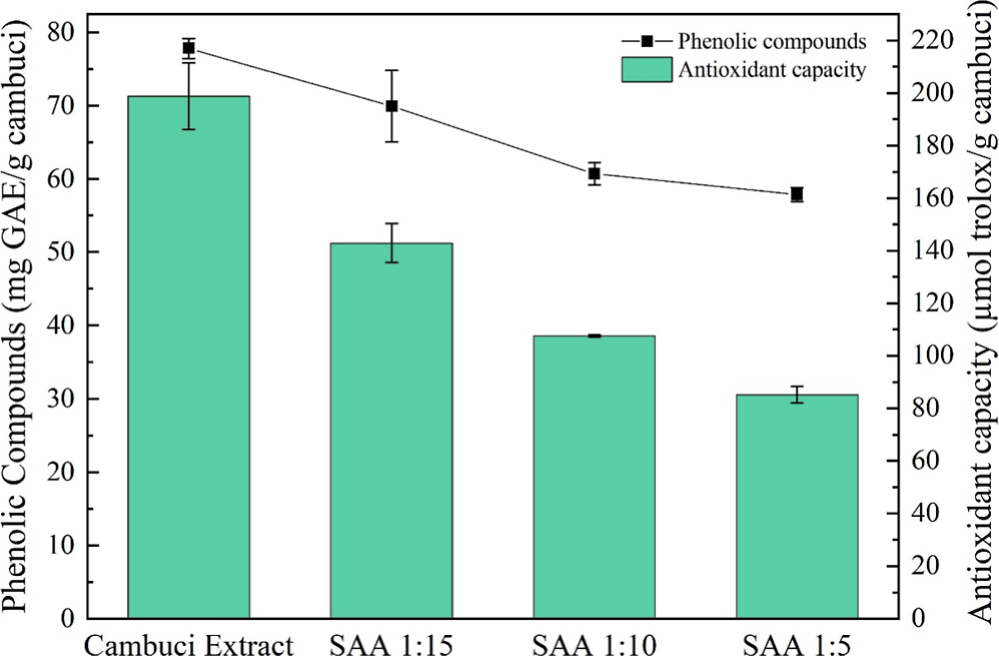
Total phenolic compounds and antioxidant capacity of each treatment. Phenolic compounds are measured in mg of gallic acid equivalent per gram of dry cambuci (mg GAE/g cambuci) and represented on the left *y*-axis. DPPH measured antioxidant capacity, scavenging capacity, and represented in micromoles of trolox per gram of dry cambuci (μmol trolox/g cambuci) and represented on the right *y* axis.

In Figure 4, it is also possible to notice not only a decrease in both phenolic compounds and antioxidant activity from the cambuci extract and the treatments but also a decrease in both factors between the treatments when the quantity of PVP is reduced. A possible reason for this behavior is that the complex extract-PVP is more thermally stable when more PVP is used. In other words, the PVP part of the complex helps to protect the extract from reactivity with the outside environment.^32^ PVP was already reported as a good preservation carrier for external environment factors such as oxygen and heat.^10,18,33,34^ Another possible reason is the encapsulation efficiency, where a larger fraction of the cambuci extract was efficiently encapsulated due to the increased availability of PVP in the system. This efficiency is lower with lower fractions of PVP, as seen in the cases of SAA 1:10 and 1:5.

### 3.3 HPLC Analysis and Compound Quantification

The quantification of AA resulted in a decrease in the concentration for the samples submitted to the SAA process. The extract presented 37.58 mg AA/g of extract, while the SAA 1:5, 1:10, and 1:15 presented, respectively, 31.94, 31.94, and 32.93 mg AA/g of extract. The SAA process resulted in approximately 85% of the original AA content for SAA 1:5 and 1:10 and 87% for SAA 1:15. Around 15% of the AA loss was observed in the experiment. This reduction may have occurred due to some losses in the process. The amount of AA obtained in the peel extract is higher than that found in the literature for pulp. Tokairin et al. reported a content of 72.16 mg AA/100 g of dry pulp.^5^ This result follows the same principle explained above for total phenolics and antioxidant activity; i.e., in the peel of some fruits, the concentration of some compounds may be higher than in the pulp.

For the gallic acid content, the results obtained were not as expected. For some reason, the gallic acid content in the SAA samples was higher than that in the extract. The extract presented 0.63 mg of GA/mg extract, while the SAA samples presented 9.79, 6.06, and 4.04 mg of GA/mg extract for SAA 1:5, 1:10, and 1:15. One possibility is that due to the low temperature of extraction, some compounds like gallotannins or ellagitannins and other secondary metabolites, present in the cambuci,^35^ that have gallic acid in the composition were preserved. During the SAA process, which occurred near 100 °C, these compounds can decompose into gallic acid, thereby increasing their yield after the process.^36^ The higher values for SAA 1:5 compared with the other SAA samples may be attributed to the lower quantity of PVP used to protect the extract, which could also have contributed to the decrease in antioxidant activity in the sample.

### 3.4 Preservation of the Bioactive Compounds over Time

After 5 months of the SAA process, the total phenolic compounds were measured by the Folin–Ciocalteu method. 99% of the active compounds of the SAA were preserved at room temperature and in artificial light. The extract showed a 30% loss of the active compounds when compared to the initial phenolic compounds. Antioxidant capacity analysis showed a similar result, with a conservation of approximately. 92% of the SAA product was preserved, whereas the liquid extract only preserved 66%. The preservation of the bioactive compounds indicates that the PVP complex protects the extract from degradation during storage.

### 3.5 Path2Green

The 12 principles of green chemistry, originally established by Anastas in 1998,^17^ have been used in the development of sustainability metrics to provide a basis for comparison between processes in terms of their associated environmental impacts. The 12 principles are Prevention, Atom Economy, Less Hazardous Chemical Syntheses, Designing Safer Chemicals, Safer Solvents and Auxiliaries, Design for Energy Efficiency, Use of Renewable Feedstocks, Reduce Derivatives, Catalysis, Design for Degradation, Real-time Analysis for Pollution Prevention, and Safer Chemistry for Accident Prevention, and their assessment is current and relevant, especially within bioeconomy and sustainable chemistry.

The Path2Green tool was chosen to carry out the sustainability assessment of the present study and to compare it with others from the literature.^12^ This tool is free for download on the Android system, and it is user-friendly. The software gets inspiration from the 12 principles from Anastas (1998) and its interface comprehends the following items and weights (settled as default): biomass (6), transport (5), pretreatment (2.5), solvent (6), scaling (5), purification (2.5), yield (4), post-treatment (2.5), energy (5), application (4.5), repurposing (6), and waste (6). The most important steps to consider for a green extraction concern the biomass (appropriate biomass helps minimize environmental footprint), the choice of solvent (preferably safe for humans and ecosystems), the repurposing (where the use of nonvirgin raw materials comes as a priority), and waste (which refers to the amount of waste generated throughout the extraction process).

To perform the analysis of the present study, the software was informed that the biomass used comes from waste, and a distance of 50 km was considered for transporting the waste biomass to the place of valorization. Physical pretreatment was applied, and the extraction methods used recommended solvents. The process was conducted in batches without purification and post-treatment steps since the powder coming from SAA is ready for use. The process is high-energy-dependent, new solvents were used, and 20% of the loss was considered since it is a two-step process (extraction and encapsulation). The obtained particles are suitable for pharmaceutical, cosmetic, and nutritional applications (three domains).

Figure 5 presents results obtained from Path2Green. The pictogram shows the general score at the center (0.266), which corresponds to the weighted average of each principle for green extraction. Additionally, a color code is used to indicate individual performance at each attribute, with red indicating poor scores, yellow indicating neutral scores, and green indicating good scores. The main opportunities for improving the methodology regard the use of pretreatment, the high-energy demand from nonrenewable sources, and the difficulty of scaling up the process. Nevertheless, the procedure in its current form is considered sustainable (since it has a positive score) and represents an opportunity to add value to a byproduct whose volume is growing.

**Figure 5:**
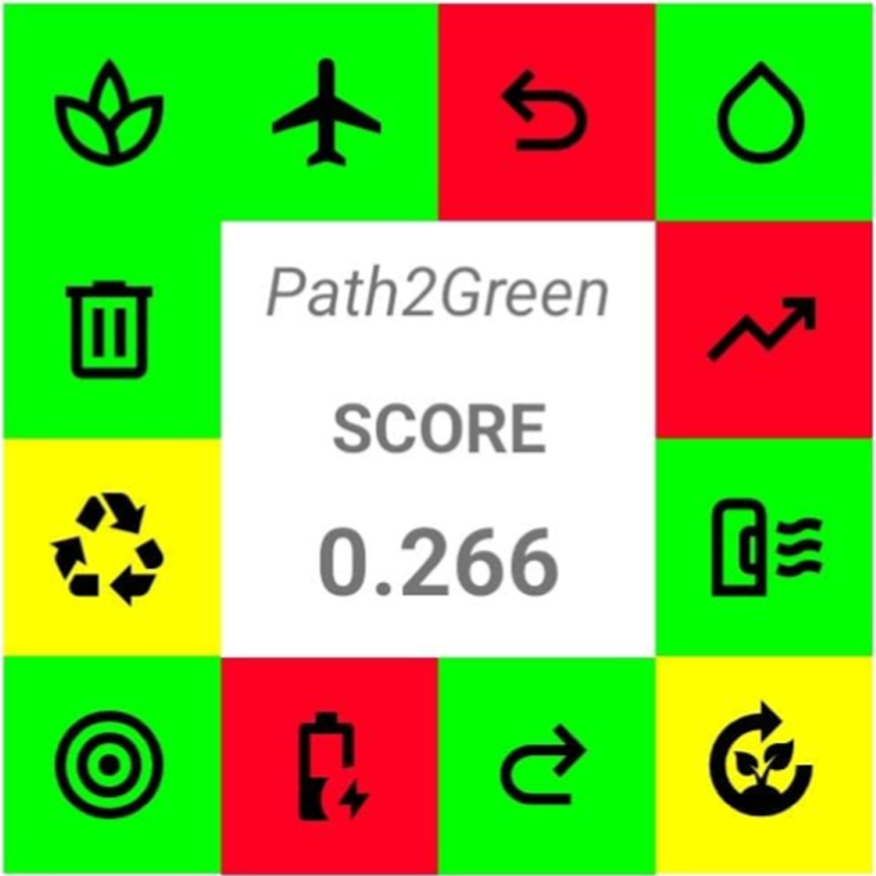
Path2Green score of the present study.

Table 3 presents the values attributed to each criterion considered in the sustainability performance analysis on Path2Green.

**Table 3:** Values Attributed to Each Criterion Considered in the Sustainability Performance Analysis on Path2Green

Criteria	present study
biomass	+1
transport (km)	50
pretreatment	–0.2
solvent	+1
scaling	–1
purification	+1
yield	0
post-treatment	+1
energy	–1
application	+0.5
repurposing	0
waste (%)	20
final score	0.266

## 4 Conclusions

Cambuci waste was successfully used to obtain a phenolic extract. Then, to preserve the biological activity, SAA was used, and it was able to produce microparticles of cambuci extract/PVP when the fraction of extract-PVP was at least 1:5. The best result in terms of phenolic compounds and antioxidant activity was with a fraction of 1:15, with antioxidant activity of 142.83 μmol trolox/g cambuci and total phenolic compounds of 69.94 mg GAE/g cambuci. The best homogeneity of particles was also found at a fraction of 1:10. Additionally, after 5 months, the encapsulated cambuci exhibited better conservation of phenolic compounds and antioxidant activity compared with the cambuci extract stored under the same conditions, indicating that PVP effectively protected the extract from environmental degradation over time.

Furthermore, using the Path2green tools, this type of project appears to better align with green chemistry principles, emphasizing the sustainability of utilizing waste as a source of active compounds and a supercritical-assisted process for encapsulation.
